# Immune checkpoint inhibitors in renal cell carcinoma

**DOI:** 10.1042/CS20160894

**Published:** 2017-10-27

**Authors:** Kirsty Ross, Rob J. Jones

**Affiliations:** 1 Department of Oncology, Beatson West of Scotland Cancer Centre, Glasgow G12 0YN, U.K.; 2 Institute of Cancer Sciences, University of Glasgow, Beatson West of Scotland Cancer Centre, University of Glasgow, Glasgow G12 0YN, U.K.

**Keywords:** cancer, immunomodulation, immunology

## Abstract

The immune system has long been known to play a critical role in the body’s defence against cancer, and there have been multiple attempts to harness it for therapeutic gain. Renal cancer was, historically, one of a small number of tumour types where immune manipulation had been shown to be effective. The current generation of immune checkpoint inhibitors are rapidly entering into routine clinical practice in the management of a number of tumour types, including renal cancer, where one drug, nivolumab, an anti-programmed death-1 (PD-1) monoclonal antibody (mAb), is licensed for patients who have progressed on prior systemic treatment. Ongoing trials aim to maximize the benefits that can be gained from this new class of drug by exploring optimal timing in the natural course of the disease as well as combinations with other checkpoint inhibitors and drugs from different classes.

## Introduction

Renal cell cancer (RCC) accounts for 2–3% of all the adult cancers [1]. The incidence of RCC has been steadily rising since the 1970s; with current U.K. incidence calculated to be 20 cases per 100000 individuals per year. In parallel, the 5-year survival rate has improved, likely as a consequence of superior surgical and medical therapeutic options along with increased detection of earlier stage tumours [2,3]. With increasing use of cross-sectional imaging, incidentally detected RCC now represents half of all the newly diagnosed RCC cases [4]. Despite these developments, a third of patients still present with locally advanced or metastatic disease and a quarter of those who present with resectable, organ-confined disease will subsequently progress to metastatic disease [5,6]. The median time to relapse post-surgical resection for local disease is 1.9 years [7]. RCC, therefore, still has a poor prognosis with 5-year survival rates for patients with locoregional and metastatic disease of 53% and 8% respectively [8,9].

**Figure 1:**
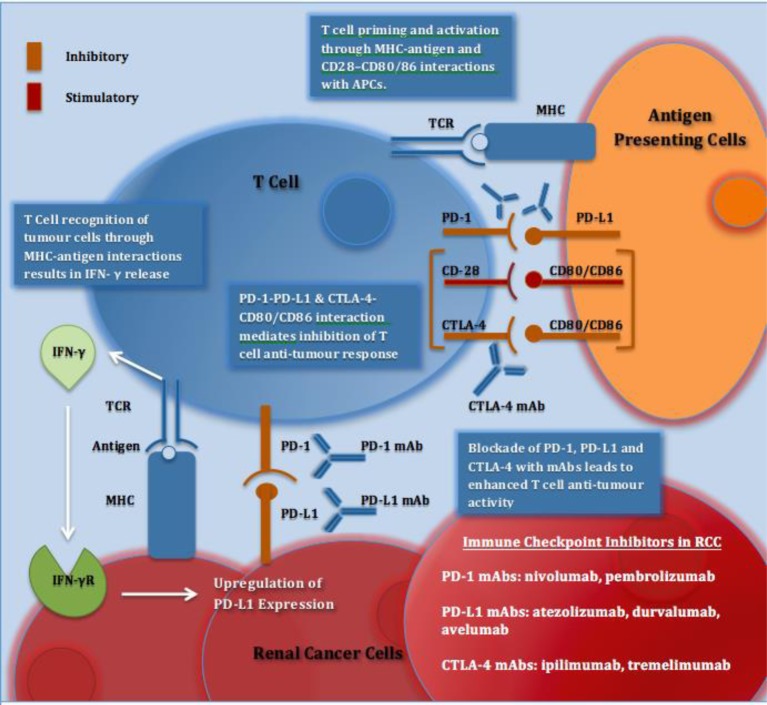
Immune checkpoints and immune checkpoint inhibitors in RCC Recognition of tumour cells and APCs via MHC–antigen interactions with TCRs activates T cells. IFN-γ released from T cells results in up-regulation of PD-L1 expression. PD-1 is expressed on activated T cells and on interaction with PD-L1 on tumour cells or APCs results in inhibition of T cell antitumour response. CTLA-4 is expressed on T cells and on interaction with its ligands CD80/CD86 on APCs, T-cell proliferation and T-cell effector function is reduced. CD28 is a co-stimulatory T-cell molecule, which has a lower affinity than CTLA-4 for their shared ligands; CD80/CD86. Blockade of PD-1, PD-L1 and CTLA-4 with mAbs stimulates an enhanced antitumour response and has shown efficacy in aRCC. Abbreviations: aRCC, advanced renal cell cancer; APC, antigen presenting cell; CD28, cluster of differentiation 28; CD80, cluster of differentiation 80; CD86, cluster of differentiation 86; CTLA-4, cytotoxic T lymphocyte associated protein 4; IFN-γ, interferon-γ; IFN-γR, interferon-γ receptor; mAb, monoclonal antibody; PD-1, programmed death-1; PD-L1, programmed death ligand 1; TCR, T-cell receptor.

Until recently, treatment options for metastatic RCC (mRCC) were limited, as it was characteristically resistant to hormonal therapy, radiotherapy and chemotherapy [10,11]. In the 1980s, multiple cytotoxic chemotherapy agents were assessed and found to have only marginal antitumour activity of less than 5–10% [12]. Over the past 20 years, significant advances have been made through greater insights into the biology of RCC and identification of drug targets such as vascular endothelial growth factor (VEGF); a key mediator in angiogenesis, platelet-derived growth factor (PDGF) and mammalian target of rapamycin (mTOR). Standard of care therapies now include orally available, multitargeted tyrosine kinase inhibitors (TKIs) such as sunitinib, pazopanib, axitinib and cabozantinib, and the mTOR inhibitors: everolimus and temsirolimus [13,14]. While these treatments have improved palliative outcomes, they are limited by both innate and acquired resistance which typically occurs within the first year of treatment [15]. Durable and complete responses (CRs) to these targeted therapies are rare and, therefore, re-exploration of the role of immunotherapy in this difficult-to-treat disease was necessary (Figure 1).

RCC has, historically, been recognized as an immune-regulated disease. Renal tumours are rich in immune infiltrates and rarely observed spontaneous regression is thought to be mediated by immune processes [16,17]. Prior to the advent of TKIs, immunotherapy with the cytokines interferon-α (IFN-α) and interleukin-2 (IL-2) were widely used palliative treatments despite modest efficacy and high burden of toxicity [15,18,19]. Therefore, when relatively more tolerable immunotherapies in the form of immune checkpoint inhibitors were developed, mRCC was one of the first solid tumours to be tested in clinical trials.

Immune checkpoint inhibitors account for the majority of immunotherapies in use today: with cytotoxic T lymphocyte (CTL) associated protein 4 (CTLA-4), programmed death-1 (PD-1) and programmed death ligand 1 (PD-L1) the principal drug targets [20]. Tumour-associated PD-L1 expression has been detected in RCC and is associated with a worse prognosis. Nivolumab, a PD-1 inhibitor, has received marketing authorization by, among other regulatory authorities, the Food and Drug Administration (FDA) and the European Medicines Agency (EMA) in the metastatic setting [21].

In this review, we will discuss pertinent background of immunotherapy in renal cancer, including IFN- α and IL-2 treatment, the biology of immune checkpoint pathways and evidence relating to current immune-checkpoint inhibitors with respect to RCC. We will conclude with some potential future developments including novel combinations and attempts to find the optimal position of immunotherapy in the disease pathway. With this shift in paradigm to reincorporation of immunotherapy in the treatment of mRCC, the sequencing and combining of treatments will also need to be explored.

## RCC

RCC is a heterogeneous disease with several histologic and molecular subtypes [22]. Subtype differentiation is an important consideration when selecting treatment as each type can indicate a very different prognoses and responses to therapy.

Clear cell RCC (ccRCC) represents the major histological subtype, accounting for approximately 75% of RCC and is often specified in the inclusion criteria for large clinical trials. ccRCC is characterized by loss of function of von Hippel–Lindau (VHL), a tumour suppressor gene. VHL is mutated in most hereditary ccRCC and in 52% of sporadic ccRCC [23]. VHL plays a central role in the oxygen-sensing pathway, targeting hypoxia-inducible factor (HIF) for degradation [24]. Mutationally inactivated VHL therefore results in intracellular accumulation of HIF and, consequently, activation of downstream pathways involved in hypoxia signalling including the production of VEGF, which stimulates angiogenesis, cell growth and survival. Other important somatic mutations found in ccRCC include PBMR1 (40%), SETD2 (15%) and BAP1 (15%), which are involved in chromatin remodelling histone methylation [23].

Non-ccRCC (nccRCC) includes two major histological subtypes, papillary RCC (type 1 and type 2) representing 10% of all RCCs and chromophobe RCC (accounting for 5% of all RCCs) [25,26], and a group of rarer histologies including collecting duct carcinoma, renal medullary carcinoma and translocation carcinoma (each approximately 1%) [23,26]. Sporadic type 1 papillary RCCs are typically present as multifocal tumours, yet demonstrate slower growth rates and metastatic potential comparative to ccRCC [27]. Type 1 papillary RCCs are closely associated with mutations in the met oncogene (*c-Met.*) On the other hand, type 2 papillary RCCs follow a more aggressive course, with greater metastatic potential and worse prognosis. Type 2 papillary tumours characteristically have alterations in the NRF2-antioxidant response element [28]. Chromophobe RCCs harbour a fairly indolent behaviour and will only rarely metastasize, with mutations found in TP53 (32% of cases) and phosphatase and tensin homologue (PTEN) (9% of cases) [29]. Mutations in the mTOR pathway have been found in chromophobe tumours (23% of cases) [30]. The collecting duct subtype is histologically and genetically similar to urothelial tumours of the upper tract. This subtype is highly aggressive, metastasizes early, and has poor response to treatment and poor prognosis. Collecting duct tumours have been associated with loss of expression of the cyclin-dependent kinase CDKN2A and SMARCB1 (INI1), a component of chromatin remodelling complex [31].

The Memorial Sloan Kettering Cancer Center (MSKCC) developed a prognostic model based on the findings from early immunotherapy clinical trials, which has been validated in the current era of TKIs. This includes five factors: poor performance status, high serum lactate dehydrogenase (LDH), high serum calcium, low haemoglobin and less than 1-year interval from diagnosis to treatment. Patients with none of these risk factors were defined as favourable-risk, those with one or two factors as intermediate-risk and those with three or more as poor-risk. The median survival for these groups was 30, 14 and 5 months respectively (*P*<0.001) [19]. The International Metastatic Renal Cell Carcinoma Database Consortium expanded the criteria for patients who had received VEGF-targeted therapies. They included four of the original five, replacing high LDH with high neutrophil count and high platelet count [32–34]. In 2013, Heng et al. [33] reported an extended validation study of over 1000 patients where median survival was 43, 22 and 8 months respectively in the favourable-, intermediate- and poor-risk groups respectively.

Treatment selection was based upon histological subtype, prognostic group and patient specific factors such as prior treatments, co-morbidities and treatment-specific toxicities.

## Cytokine immunotherapy in RCC

The cytokines IFN-α and IL-2 were at one stage the only systemic therapies to demonstrate significant clinical benefit in metastatic renal cancer. Routine use ceased owing to their high toxicity and the arrival of TKIs such as sunitinib and pazopanib.

### IFN-α

IFN-α is a pleiotropic cytokine with immunomodulatory, antiviral, antiproliferative and anti-angiogenic properties which can induce the differentiation of monocytes into highly activated dendritic cells (DCs.) These DCs (IFN-DCs) are particularly effective in recognizing complex antigens and inducing T- and B-cell immunity and thus participate in the generation of antitumour T-cell immunity [35].

In 1993, Minasian et al. [125] reported an overall response rate (ORR) of 10% in 159 patients with mRCC treated with IFN-α. The median duration of response (DOR) was 12.2 months and the median overall survival (OS) was 11.4 months. Subsequent studies have reported ORRs at approximately 15% (range: 0–29%) [36]. A survival benefit was demonstrated in a randomized phase III trial comparing INF-α plus vinblastine with vinblastine monotherapy (median OS: 67.6 and 37.8 weeks respectively (*P*=0.0049)) [37]. IFN therapy was often poorly tolerated with influenza-like side effects including fatigue, fever, chills, myalgia and depression being common and often dose limiting [38].

### IL-2

IL-2 is a naturally occurring cytokine, which plays a central and multifaceted role in the immune system [39]. IL-2 was first identified in 1976 as a T-cell growth factor, a 15-kDa glycoprotein produced primarily by T-helper cells, demonstrated to have potent capacity to enhance *in vitro* T-cell proliferation and differentiation [40]. Ligation of IL-2 with the IL-2 receptor, which is normally highly expressed on activated T cells, results in proliferation and differentiation of B and T cells and stimulation of a cascade of cytokines, including various interleukins, interferons and tumour necrosis factors (TNFs) [12]. The anti-tumour effect of IL-2 is mediated by its ability to cause proliferation of natural killer cells (NK), lymphokine-activated killer cells (LAK) and other cytotoxic cells [12]. IL-2 receptor activation initiates signal transduction through the Janus kinase 3 (JAK3), signal transduction activator of transcription 5 (STAT5), mitogen-activated protein kinase (MAPK) and phosphatidylinositol 3-kinase (PI3K) pathways. Activation of these pathways effects gene expression altering cellular growth, death and immune function. While IL-2 is required to mount and sustain adaptive T-cell responses, it is now also understood that it plays a critical role in immune regulation via its effects on regulatory T cells (Treg cells) [39].

In 2000, Fisher et al. [41] reported long-term survival results for high dose (HD) IL-2 treatment in 255 patients with mRCC treated in seven phase II clinical trials. Recombinant IL-2 600000 or 720000 IU/kg was administered by 15-min intravenous infusion every 8 h for up to 14 consecutive doses over 5 days as clinically tolerated with maximal support. A second cycle of treatment was scheduled after 5–9 days of break from treatment, and courses could be repeated every 6–12 weeks in stable or responding patients. ORR was 15% with 7% experiencing CR. Median response duration for all objective responders was 54 months (range: 3 to >131 months). Klapper et al. [18] reported an analysis of 259 mRCC patients treated with HD IL-2 alone between 1986 and 2006. ORR was 20% with 8.8% experiencing CR. At the time of last follow-up, all partial responders had developed disease progression but only 4 out of 23 complete responders had experienced disease recurrence. A lower MSKCC prognostic factor score (*P*=0.02) was found to be the variable most associated with response [18]. In a retrospective analysis of pathology specimens obtained from 163 patients who had received IL-2 therapy, the response rate to IL-2 was 21% for patients with ccRCC histology compared with 6% for patients with nccRCC histology [42].

HD IL-2 received FDA approval for mRCC based upon results reporting durable responses [41]. Unfortunately, the major limitation of HD IL-2 was the high incidence of severe toxicity. Grades 3/4 toxicities developed in most patients treated with HD IL-2 and approximately 4% of patients died of treatment-related toxicity. The most common toxicities resembled the symptoms of septic shock, including hypotension, which occurred in 96% of patients (grades 3/4, 74%) [5]. HD IL-2 could therefore only be administered in hospitals which could provide the appropriate level of medical care to support these severe cardiovascular toxicities [15].

In attempt to circumvent this issue, several clinical trials were performed investigating variable IL-2 regimens involving lower doses either alone or in combination with interferon but failed to show comparable activity [43]. In an attempt to improve the therapeutic index of IL-2, the Cytokine Working Group (CWG) designed and conducted the HD IL-2 “Select” trial. The primary objective of this prospective study was to evaluate whether they could identify a group of patients with advanced RCC and “good” predictive features who were significantly more likely to respond to HD IL-2 than a historical, unselected patient population [44,45]. The trial failed to validate the proposed new tool or other potential predictive biomarkers such as carbonic anhydrase IX (CAIX), SNP status, plasma VEGF or fibronectin levels. The trial did, however, report durable remissions and prolonged survival in both “good” and “poor-risk” patients, which exceeded historical results: independently assessed ORR was 25% including 3 (2.5%) CRs among 120 patients. Thirteen (11%) remained progression free at 3 years and the median OS was 42.8 months. In addition, tumour PD-L1 expression by immunohistochemistry (IHC) appeared to warrant future investigation. Through gene expression profiling of tumour specimens, Pantuck et al. [46] were able to identify a set of 73 genes whose expression distinguished complete responders from non-responders after IL-2 therapy. Complete responders to IL-2 were reported to have a signature gene and protein expression pattern that included CAIX, PTEN and chemokine C-X-C receptor 4 (CXCR4) [46].

## Immune checkpoint pathways

Immune checkpoints consist of multiple co-stimulatory and inhibitory interactions, which sustain self-tolerance and modulate physiological immune responses. The amplitude, duration and quality of a response are initiated through antigen recognition by the T-cell receptor (TCR), then ligand–receptor interactions [20]. This modulation is to optimize targeting of unwanted cells and the preservation of normal tissue (i.e. to inhibit autoimmunity). Immune checkpoint pathways such as PD-1/PD-L1 and CD28/CTLA-4 are co-opted by cancer, resulting in altered expression of proteins to assist in the masking of cancer cells from immune surveillance and thus to evade immune destruction [47,48].

Cancer cells and immune cells mutually influence each other, allowing cancer to escape immunosurveillance and immune attack. The equilibrium between tumour and immune system is complex with immune checkpoint exploitation is only one mechanism of many. Intrinsic mechanisms in tumour cells, such as down-regulation of MHC class I and II molecules and/or tumour-associated antigens (TAAs), result in reduced presentation and subsequent targeting by immune effector mechanisms [49]. Cancer cells also secrete immunosuppressive cytokines such as interleukin-10 (IL-10) and transforming growth factor-β (TGFβ) [50,51]. Furthermore, tumour infiltration by tumour-associated macrophages (TAMs) and Tregs has been correlated with reduced survival. It is hypothesized that TAMs may drive the infiltrating T cells towards a more regulated phenotype at the expense of protective effector functions [52].

T cells have several antitumour competencies: they can recognize peptides on the surface of cellular compartments and kill antigen-expressing cells (by CD8^+^ effector T cells, also known as CTLs) and co-ordinate complex immune interactions (by CD4^+^ helper T cells) [48,53]. Agents targeting the immune checkpoint pathways therefore aim to amplify the antigen-specific T-cell responses. In general, it is soluble and membrane-bound receptor–ligand immune checkpoints that are the most suitable targets for drug delivery – with agonist antibodies for co-stimulatory pathways or antagonist antibodies for inhibitory pathways [47]. An important consideration is that, in comparison with most oncological antibodies, many immune checkpoint targeting agents target lymphocyte receptors or ligands to augment endogenous antitumour activity rather than targeting tumour cells directly. This may have important implications for acquired drug resistance.

### The CD28/CTLA-4 system

In 1996, Leach et al. [54] made the pivotal observation that blocking CTLA-4 could mediate tumour regression in murine models. This work led to the development of anti-CTLA-4 antibodies, which have become a standard of care for metastatic melanoma [55,56]. CTLA-4 is an inhibitory receptor expressed exclusively on T cells (both CD4^+^ helper T cells and CD8^+^ cytotoxic T cells). CTLA-4 is predominantly expressed on CD4^+^ helper cells; therefore enhanced CD8^+^ responses in anti-CTLA-4 treated patients are likely to be an indirect effect related to activation of CD4^+^ cells [57]. In cancer, CTLA-4/CD28 engagement down-modulates helper T-cell activity and enhances Tregs immunosuppressive activity [58]. *In vivo* studies with CTLA-4-deficient mice have shown that they develop profound autoimmunity and succumb to lymphoproliferative disease by 4 weeks of age [59,60]. In humans, *CTLA-4* gene polymorphisms have been associated with the onset of several autoimmune conditions including autoimmune hypothyroidism and type 1 diabetes [61]. CTLA-4 inhibition has two main actions – inhibition of peripheral T-cell tolerance resulting in autoimmunity and activation of antitumour immunity [47].

CTLA-4s main ligands CD80 and CD86 are expressed on antigen-presenting cells (APCs) (such as DCs and monocytes) but not on non-haematologic tumour cells. Given the location of ligand expression, the suppression of antitumour immunity by CTLA-4 is therefore considered to act, principally, in secondary lymphoid organs where T-cell activation occurs [20]. Studies have also reported a possible direct inhibitory role of CTLA-4 on CD8^+^ T cells [57]. CTLA-4 also engages with the TCR ‘stop signal’, supporting the maintenance of the immunological synapse to allow serial interactions between TCRs and APCs [62]. Naïve and resting memory T cells express CD28 but not CTLA-4. At antigen recognition, CTLA-4 will however be quickly transported to the cell membrane from intracellular stores to allow negative feedback. This usually occurs within an hour after antigen recognition [20]. CTLA-4 has also been reported to enhance the suppressive action of Treg cells. Treg cells are focused in tumour tissues and inhibit effector T-cell activity thus inhibit antitumour immunity locally [20,63].

In humans, anti-CTLA-4 therapy activates expression of stimulatory markers on T cells and can result in inflammatory side effects. The fully human IgG1 anti-CTLA-4 monoclonal antibody (mAb) ipilimumab (Bristol-Myers Squibb) and tremelimumab (AstraZeneca/MedImmune), a fully human IgG2 anti-CTLA-4 mAb are the leading CTLA-4 targeted immune checkpoint inhibitors [55,64]. Ipilimumab received US-FDA and EMA’s approval in 2011 for advanced, unresectable melanoma where it is now established as a standard of care.

### The PD-L1/PD-1 system

PD-L1 is highly expressed in tumour cells and tumour-infiltrating lymphocytes (TILs) within the tumour microenvironment [65]. In renal cancer, PD-L1 (also known as B7-H1, CD274) expression on either tumour cells or TILs in primary tumours correlates with a worse prognosis, with reduced OS compared with PD-L1 negative tumours [66–68]. PD-L1 seems to be the major ligand in solid tumours and PD-1’s alternative ligand, PD-L2, within subsets of B-cell lymphoma [69,70]. PD-1 is expressed more diffusely than CTLA-4, being present on other, activated, non T-lymphocyte subsets, such as B cells and NK cells, reducing their lytic capacity. As with CTLA-4, PD-1 is not present on resting naïve or memory T cells, yet is expressed at antigen recognition and TCR engagement [71]. PD-1 expression on activated T cells takes longer to surface than CTLA-4 as it requires transcriptional activation, usually taking approximately 6–12 h. Chronic antigen exposure can produce persistently elevated PD-1 expression that culminates in an exhausted antigen-specific T-cell colony. This state has been reported in both mice and humans and found to be partially reversible with PD1 pathway blockade [72]. Mouse models with knockout of PD-1 and its known ligands result in mild phenotypes, with organ-specific inflammation, which is a stark contrast with the CTLA-4 knockout models where death occurs by 4 weeks of age [73].

PD-1 has a pivotal physiological role in T-cell inhibition in the peripheral tissues during inflammatory reactions, therefore reducing autoimmunity and ‘collateral damage’. With up-regulation of PD-1 and PD-L1 expression in cancerous tissues, tumours develop an immune resistant phenotype within the tumour microenvironment. There are several processes, including adaptive immune resistance, which involves enhanced ligation of PD-L1 to PD-1 on antigen-specific CD8^+^ T cells, which inhibit cytotoxic activity against cells presenting tumour antigens. PD-1 activation directly inhibits TCR-mediated effects and increases T-cell migration within tissues, thus reducing the time that a T cell has to evaluate the surface of cells for the presence of MHC–peptide epitopes. With reduced time for surveying, T cells may fail to identify cells expressing lower levels of MHC–peptide complexes – thus cancer cells could evade immune surveillance and immune destruction. PD-1 signalling exerts major effects on cytokine production by T cells, inhibiting production of interferon-γ (IFN-γ), TNF-α and IL-2 [74]. PD-1 can also inhibit T-cell proliferation and inhibit the up-regulation of Bcl-xL, an anti-apoptotic protein.

PD-L1 and PD-L2 are expressed by tumour cells and infiltrating leucocytes within the tumour microenvironment. PD-L1 is expressed on haematopoietic cells and epithelial cells – stimulated by IFN-γ, the inflammatory cytokine, which is produced by activated T and NK cells [74]. PD-L2 is expressed on activated DCs and some macrophages. PD-L2 expression is induced by interleukin-4 (IL-4) and to a lesser extent by IFN-γ [69].

Targeted therapies against PD-1 receptor and its ligand PD-L1 have demonstrated impressive response rates with minimal toxicity in several solid malignancies [66]. Early exploratory studies found that melanoma, ovarian and lung cancer biopsies had high PD-L1 expression levels and multiple other solid tumours have subsequently been found to have up-regulated expression. Two mechanisms are understood to account for PD-L1 up-regulation: innate or tumour cell intrinsic and adaptive immune resistance, which can coexist in a single tumour microenvironment [48].

There are a number of drugs targeting either PD-1 or PD-L1. Notably, nivolumab and pembrolizumab (anti-PD-1) are licensed to treat a number of tumour types including non-small-cell lung cancer (NSCLC), melanoma, head and neck cancer, urothelial bladder cancer, RCC and Hodgkin’s lymphoma. Atezolizumab (anti-PD-L1) is licensed in the treatment of advanced urothelial cancer. In addition, avelumab and durvalumab (anti-PD-L1) are in late-stage clinical development in a number of indications [75].

### Radiological response – immune-related response evaluation criteria in solid tumours

In contrast with chemotherapy and TKIs, responses to CTLA-4 inhibitors and similarly, to PD-1/PD-L1 inhibitors may be delayed (can be up to 6 months after treatment) [20]. Radiologically, tumour sites have been observed to increase in size prior to regression. This is presumed to be due to initial immune infiltration causing early tumour swelling. This phenomenon has been termed as pseudoprogression [76]. Given these fluctuations, an immune-related Response Evaluation Criteria in Solid Tumours (ir-RECIST) has been developed to create a uniform approach to response/progression assessment with these drugs [77].

### Immune-related adverse events

Immune checkpoint inhibitors are also associated with a spectrum of treatment related adverse events (AEs), which differs from that seen in other classes of drug. An augmented immune response driven by T-cell activation can result in potential autoimmune-related inflammation of normal tissues. The most common AEs are fatigue, rash, nausea, pruritus and diarrhoea [78]. Less common events include hepatitis, colitis, pneumonitis, nephritis, endocrinopathies (such as hypophysitis, hypo/hyperthyroidism) and neurological conditions such as Guillain–Barré syndrome (GBS) [78]. Side effects are generally manageable with supportive measures and corticosteroids in some cases; they can, however, rarely, be fatal. Patient and staff education is therefore crucial and a high index of suspicion regarding immune-related AEs (irAEs) should be maintained for all the patients on immune checkpoint inhibitors.

## Current evidence in RCC

Over recent years, a multitude of clinical trials has investigated immune checkpoint inhibitors, principally: PD-1, PD-L1 and CTLA-4 mAbs. We summarize data in advanced RCC (aRCC) from pivotal trials in Table 1.

**Table 1:** Single agent anti-PD-1, anti-PD-L1 and anti-CTLA-4 studies in aRCC

Trial	Trial summary	Number of patients (RCC)	Dose of trial drug	ORR (%)	Median progression- free survival (PFS) (months)	Median OS (months)	Immune-related G3/4 toxicities
**Nivolumab (fully human IgG4 anti-PD-1 mAb)**							
NCT00730639 McDermott et al. [87]	Phase I study in patients with advanced solid tumours with a RCC cohort	296 (34)	1 mg/kg	24%	NR	All patients: 22.4; 4-year survival rate: 38%	18%
			10 mg/kg	31%			
			Every 2 weeks				
NCT01354431 Motzer et al. [82]	Phase II study in aRCC. Patients randomly assigned in one of three dose groups	168 (168)	0.3 mg/kg	20%	2.7	18.2	11% (*n*=19)
			2 mg/kg	22%	4.0	25.5	
			10 mg/kg	20%	4.2	24.7	
			Every 3 weeks			Four-year survival rate: 29%	
Checkmate 025 NCT01668784 Motzer et al. [83]	Randomized, open-label phase III study of nivolumab compared with everolimus in patients with aRCC who had received ≥1 prior regime of anti-angiogenic therapy	Nivolumab (406)	3 mg/kg	25%	4.6 m	25	19% (76/406)
			Every 2 weeks			Improved health related QoL	All G3/4 AEs 20%
		Everolimus (415)	10 mg OD	5%	4.4	19.6 (*P*=0.002)	NR
							All G3/4 AEs: 37%
**Atezolizumab (human IgG1 anti-PD-L1 mAb)**							
NCT01374842 McDermott et al. [88]	Phase Ia dose-escalation and dose-expansion study with a RCC cohort.	(70)	10, 15, 20mg/kg every 3 weeks				All G3/4 AEs: 17%
		ccRCC 63		15%	5.6	28.9	4%
		nccRCC 7		0%	NR	NR	NR
**BMS-936559, MDX-1105 (fully human IgG4 anti-PD-L1 mAb)**							
NCT0072966 Brahmer et al. [89]	Phase I dose-escalation and dose-expansion study in patients with advanced solid tumours including an RCC cohort	207 (17)	10 mg/kg	12%	Stabilization of disease at 24 weeks in 41%	NR	All G3/4 AEs: 5%
**Ipilimumab (fully human IgG1 anti-CTLA-4 mAb)**							
Yang et al. [86]	Single institution, phase II study of patients with mRCC. Patients were allowed to have had prior treatment with IL-2	Cohort A (21)	3 mg/kg loading	5%	NR	NR	Both groups: 33%
			Then 1 mg/kg				Colitis: 28%
			Every 3 weeks				Hypophysitis: 5%
		Cohort B (40)	3 mg/kg all doses	12.5%	NR	NR	
			Every 3 weeks				
**Tremelimumab (fully human IgG2 anti-CTLA-4 mAb)**							
Ribas et al. [90]	Phase I dose escalation study of patients with advanced melanoma, RCC or colorectal cancer (CRC)	39 (4)	MTD: 15 mg/kg	NR	NR	NR	

Abbreviations: MTD, maximum tolerated dose; NR, not reached.

### PD-1 pathway inhibitors

Nivolumab (Bristol-Myers Squibb; New York, NY, U.S.A.) is a fully human monoclonal IgG4 mAb that is specific for PD-1 and has received FDA and EMA approval in NSCLC, RCC and head and neck cancers [79].

The first in-human phase I study of nivolumab (MDX-1106/BMS-936558/ONO-4538) was conducted in 39 patients with advanced metastatic melanoma, colorectal cancer (CRC), castrate-resistant prostate cancer, NSCLC or RCC. Brahmer et al. [80] published their findings from this in 2010 – demonstrating tumour responses in melanoma, RCC and CRC and a favourable toxicity profile. In response to this, 296 patients with various solid malignancies, including 34 patients with RCC, were enrolled in a phase I multiple-dose basket trial. Objective responses were reported in 29% (10/34) of patients with RCC. Responses were seen at both doses of nivolumab explored in the present study (1.0 and 10.0 mg/kg). Another nine patients (27%) had stable disease for 24 weeks or more. The median progression-free survival (PFS) for RCC patients in this trial was 7.3 months, with 1-year PFS rate of 35% and 2-year PFS rate of 12% [81].

In a subsequent phase II trial, 168 patients with progressive, advanced or metastatic ccRCC were randomized to receive doses of 0.3 mg/kg (*n*=60), 2.0 mg/kg (*n*=54) or 10.0 mg/kg (*n*=54) of nivolumab every 3 weeks until disease progression or unacceptable toxicity. The median PFS (the primary end point) was 2.7 months in the 0.3 mg/kg group, 4.0 months in the 2 mg/kg group and 4.2 months in the 10 mg/kg group [82]. The median OS was 18.2, 25.5 and 24.7 months respectively with ORRs of 20, 22 and 20%. Thirty-five patients (54% of responders) had responses lasting for at least 12 months. Grade 3/4 (G3/4) treatment related AEs were present in 5% of patients at the 0.3 mg/kg dose, 17% of patients at the 2 mg/kg dose and 13% of patients at the 10 mg/kg dose. Discontinuation of treatment due to AEs was necessary in 2, 11 and 7% respectively. The present study concluded that nivolumab was well tolerated and demonstrated sufficient activity to justify a randomized, phase III trial [82].

Motzer et al. [83] reported results from the open-label phase III CheckMate 025 trial in 2015. Eight hundred and twenty one patients with advanced ccRCC who had received one or two prior regimens (including at least one targeting VEGFR) were randomized to everolimus or nivolumab 3 mg/kg fortnightly. The primary end point was OS. The hazard ratio for death was 0.73 (98.5% CI: 0.57–0.93; *P*=0.002), which met the prespecified criterion for superiority (*P*≤0.0148). The median OS gain was 4.4 months (25.0 months for the nivolumab group and 19.6 months for the everolimus group, *P* = 0.002). The ORR was greater with nivolumab than with everolimus (25 compared with 5%; odds ratio: 5.98 (95% CI: 3.68–9.72); *P*<0.001) and the median PFS was 4.6 months (95% CI: 3.7–5.4) with nivolumab and 4.4 months (95% CI: 3.7–5.5) with everolimus (hazard ratio: 0.88; 95% CI: 0.75–1.03; *P* =0.11). G3/4 AEs occurred in 20% of patients receiving nivolumab compared with 37% in the everolimus group. The most common G3/4 toxicity in the nivolumab group was fatigue (2%). In the everolimus group, 9% had G3/4 anaemia, 5% G3/4 hyperglycaemia, 4% G3/4 stomatitis, 3% G3/4 fatigue and 1% G3/4 rash. PD-L1 expression (≥1 or <1%) was not predictive of OS [83]. Using a Quality of Life (QoL) questionnaire tailored for renal cancer (Functional Assessment of Cancer Therapy in Kidney Symptom Index-Disease Related Symptoms (FKSI-DRS)), the mean change in baseline in the nivolumab group increased over time and differed significantly from the everolimus group at each assessment through to week 76 (*P*<0.05) [84]. An updated report after 24 months follow-up found median OS benefit increased to 6.3 months (hazard ratio =0.73; *P*=0.0006) [85]. Thus, nivolumab was shown to improve efficacy outcomes for patients with metastatic ccRCC with a manageable toxicity profile and better QoL when compared with everolimus.

### Anti-CTLA-4 mAbs

In 2007, Yang et al. [86] reported phase II results of ipilimumab administration in a cohort of 62 patients with mRCC. With an ORR of just 10% and 33% of patients experiencing a G3/4 immune-mediated toxicity, ipilimumab was not taken forward into further trials in RCC at that time. Notably, of the the 20 patients with significant immune-related toxicity the response rate was 30%, yet among the 41 patients free of such toxicity was 0% (*P*=0.0007; both cohorts combined). Within the cohort of patients who responded some had significant durable responses [86].

### Anti-PD-L1 mAbs

Results on the efficacy of PD-L1 inhibitors in aRCC are more limited than PD-1 inhibitors due to their comparative earlier stage of drug development.

BMS-936559 (MDX-1105; Bristol-Myers Squibb) is a fully human IgG4 mAb, which binds to PD-L1. A first in-human phase I trial administered BMS-936559 twice weekly to 207 patients with solid cancers, 17 of whom had aRCC. A maximum tolerated dose (MTD) was not reached. G3/4 toxicities were reported in 19 of 207 patients (9%), with immune-mediated causality potentially in 10 of 207 (5%.) Objective response was observed in 2 of 17 patients (12%); with durations of response being 4 months and 17 months. Seven of the seventeen patients (41%) had stable disease for more than 24 weeks. Objective responses were also demonstrated in patients with ovarian cancer, melanoma and NSCLC [89]. The development of BMS-936559 in solid cancers has not been pursued. This drug is currently being investigated in HIV-infected patients and a recent phase I reported that a single low-dose infusion appeared to enhance HIV-1 specific immunity in a subset of participants [91].

Atezolizumab (MPDL3280A (Genentech; South San Francisco, CA, U.S.A.)) is a human IgG1 mAb, with an engineered fragment crystallizable (Fc) domain designed to inhibit antibody-dependent, cell-mediated cytotoxicity, and therefore avoid cytotoxic activity against activated T cells expressing PD-L1. A phase I trial administered atezolizumab every 3 weeks to 171 patients with advanced solid cancers, including 55 patients with RCC [92]. G3/4 AEs were observed in 22 of 171 patients (13%), G3/4 irAEs occurred in 4 patients (2%.) Forty-seven RCC patients were evaluable for efficacy, with objective responses in six patients (13%), this included one patient with nccRCC. An additional 32% of the RCC cohort had stable disease for more than 24 weeks [93].

Atezolizumab was also investigated in phase Ia dose-escalation and dose-expansion trial of 70 patients with mRCC (ccRCC, *n*=63 and nccRCC, *n*=7) on a 3-week schedule. Median OS was 28.9 months, PFS: 5.6 months and ORR: 15%. G3 treatment related and irAEs occurred in 17 and 4% of patients respectively. There were no grade 4 or 5 events. IrAEs were reported in 30 patients (43%), with the most common being grade 1 rash (20%) and grade 2 hypothyroidism (10%.) Interestingly, patients with poor prognostic features such as poor MSKCC prognostic status, high Fuhrman grade and/or sarcomatoid features demonstrated a higher ORR. The ORR for the 16 patients with grade 4 tumours was 25% and ORR was 33% for those with a component of sarcomatoid histology. This trial also investigated potential biomarkers including a panel of 94 circulating biomarkers assessed at baseline and on day 1 of cycle 3 in 63 patients to assess for association with OS. Plasma VEGFA was reported to decrease in responders and stable in patients with stable or progressive disease. In addition, on treatment reduction in acute-phase proteins, including ferritin, complement C3, vitamin D-binding protein and macrophage inflammatory protein-1α were significantly associated with longer OS. Lower baseline levels of multiple acute-phase proteins, including von Willebrand factor, serum amyloid P component, α-1-antitrypsin and fibrinogen (negative prognostic factors in RCC) were also associated with longer OS. RNA from archival tumour biopsies was analysed for markers of tumour immune biology. A higher ratio of effector T cells to Treg cells (represented by *FOXP3* expression was associated with atezolizumab response (*P*=0.035) [88].

## PD-L1 expression

The role of PD-L1 expression, within tumour cells or immune cells within the tumour microenvironment, as a predictive biomarker in aRCC remains unclear. Data mirror the conflicting results seen across other tumour types, with no validated correlation between PD-L1 expression and response to immune checkpoint inhibitors [94].

In CheckMate 025, 90% of patients in the nivolumab treatment arm (370 of 410 patients) had quantifiable tumour PD-L1 status. This was categorized into PD-L1 expression <1% or ≥1%. Patients experienced survival benefit from nivolumab irrespective of PD-L1 expression. Subgroup analysis of the nivolumab cohort demonstrated higher response rates in patients with tumours with  ≥1% PD-L1 expression. These patients however, had a lower median OS (median OS: ≥1 compared with  <1% PD-L1 expression, 21.8  months (95% CI: 16.5–28.1) compared with 27.4  months (95% CI: 21.4–NR) respectively), potentially indicating the more aggressive nature of tumours expressing PD-L1 in aRCC [83]. Subgroups analysis of phase I data of atezolizumab in aRCC demonstrated that patients with lower PD-L1 expression (<1%) associated with lower PFS and OS [88]. A recent hypothesis generating study sought to investigate underlying mechanisms which resulted in failure of PD-1-targeted therapies in patients with aRCC expressing PD-L1. RNA was isolated from PD-L1 positive tumour biopsy regions, before undergoing gene expression analysis and whole genome microarray. The study reported a potential association between genes involved in metabolic and solute transport functions such as UGT1A family members and treatment failure in patients with PD-L1-positive RCC. For biopsies from responding patients there was overexpression of some important immune markers involved in CD4^+^ T-cell differentiation and leucocyte differentiation [95].

Further studies are necessary to investigate the role of PD-L1 expression as a predictive biomarker in aRCC.

### Combination regimens

While the results from Checkmate 025 were very encouraging, only 1% of patients receiving nivolumab achieved CRs, and 31% of patients achieved durable responses greater than 12 months [83]. Due to the complex and dynamic nature of tumour immune response, there is a clear rationale in utilizing combination treatments to enhance antitumour effect [96]. Combination treatment strategies with other checkpoint inhibitors or angiogenesis inhibitors are currently being investigated in multiple trials in both treatments – naïve and previously treated aRCC patients and also in the neoadjuvant and adjuvant settings (Table 2 includes some of these). Recent evidence suggests that anti-angiogenesis therapies have immunomodulating effects, such as promoting intratumoral T-cell infiltration or increasing tumour antigenicity, which could potentiate the effect of immune checkpoint inhibitors [97].

**Table 2:** Ongoing combination phase I-III clinical trials with immune checkpoint inhibitors in aRCC

Trial	Phase	Trial summary	Population	Trial status	Estimated study completion date
Checkmate 016 NCT01472081 [100,101]	I	Nivolumab + ipilimumab	Treated and untreated aRCC	Study ongoing; not recruiting	June 2018
		Nivolumab + sunitinib			
		Nivolumab + pazopanib			
NCT02210117 [102]	Early phase I	Experimental arm A – nivolumab	Neoadjuvant pilot – mRCC (clear cell) who are eligible for cytoreductive nephrectomy, metastasectomy or post-treatment biopsy. Treated and untreated	Study currently recruiting	November 2019
		Experimental arm B – nivolumab + bevacizumab			
		Experimental arm C – nivolumab + ipilimumab			
NCT02348008 [103]	I/II	Pembrolizumab + bevacizumab	In first- and second-line treatment for aRCC (clear cell)	Study ongoing; not recruiting	March 2018
		Arm A – phase Ib – dose escalation			
		Arm B – phase II			
Keynote 018 NCT02014636 [104]	I/II	Pembrolizumab + pazopanib	Treatment naïve patients with aRCC	Study ongoing; not recruiting	February 2019
		Part 1 – dose escalation			
		Part 2 – randomized three arm			
NCT02133742 [105]	Ib	Pembrolizumab + axitinib	Treatment naïve aRCC (clear cell)	Study ongoing; not recruiting	April 2018
		Experimental: dose-finding and dose-expansion phase			
Keynote 29 NCT02089685 [106]	I/II	Pembrolizumab + pegylated IFNα-2b (PEG-IFN)	Previously treated aRCC (clear cell) and treatment naïve or treated advanced melanoma	Currently recruiting patients	June 2020
		Pembrolizumab + ipilimumab			
JAVELIN Renal 100 NCT02493751 [107]	Ib	Avelumab + axitinib	Treatment naïve aRCC (clear cell)	Currently recruiting patients	February 2019
		Experimental: dose-finding + dose-expansion phase			
NCT0197583 [108]	I	Durvalumab (MEDI4736) + tremelimumab IV	Patients with advanced solid tumours; RCC, colorectal, breast, ovarian and cervical	Ongoing but not recruiting	October 2017 (Primary end point)
NCT01984242 IMmotion150 [109]	II	Multicentre randomized, open-label study	Treatment naïve aRCC (clear cell +/or sarcomatoid)	Ongoing but not recruiting	August 2019
		Experimental arm A – atezolizumab + bevacizumab			
		Experimental arm B – atezolizumab			
		Comparator arm C – Sunitinib			
Checkmate 214 NCT02231749 [110]	III	Randomized, open-label study	Treatment naïve aRCC	Ongoing but not recruiting	September 2019
		Experimental: arm A: nivolumab + ipilimumab			
		Active comparator: arm B: sunitinib 50 mg			
Keynote-426 NCT02853331 [111]	III	Randomized, open-label study	Treatment naïve aRCC (clear cell with or without sarcomatoid features)	Currently recruiting	January 2020
		Experimental arm – pembrolizumab + axitinib			
		Comparator arm – sunitinib monotherapy			
IMmotion151 NCT02420821 [112]	III	Multicentre, randomized, open-label study	Treatment naïve aRCC (clear cell and/or component of sarcomatoid)	Ongoing but not recruiting	July 2020
		Experimental: atezolizumab + bevacizumab			
		Active comparator: sunitinib			
JAVELIN Renal 101 NCT02684006 [113]	III	Experimental: avelumab + axitinib	Treatment naïve aRCC (clear cell)	Currently recruiting patients	September 2020
		Active comparator: sunitinib			

Abbreviations: Atezo, atezolizumab; BD, twice daily; Bev, bevacizumab; D1, day 1; Ipi, ipilimumab; Nivo: nivolumab, OD, once daily; Pembro, pembrolizumab; QD, four times daily.

### Combination regimens – immune checkpoint inhibitors

Checkmate 016 was a three-armed phase I study, exploring different dose combinations of nivolumab and ipilimumab in previously treated or treatment-naïve mRCC patients (*n*=44). The provisional analysis of the secondary end points has reported an ORR of 43% in patients treated with nivolumab 3 mg/kg and ipilimumab 1 mg/kg every 3 weeks (nivo3 and ipi1) (four treatments) followed by nivolumab every 2 weeks until progression (*n*=21). This regime however incurred G3/4 treatment related AEs in 38% (nivo3 + ipi1) and 62% (nivolumab 1 mg/kg plus ipilimumab 3 mg/kg, (nivo1 + ipi3)) of patients. The most common of these were gastrointestinal and hepatic, including elevated lipase (15 compared with 28%), elevated ALT (4 compared with 21%), diarrhoea (4 compared with 15%), elevated AST (4 compared with 13%), and colitis (0 compared with 15%). The median DOR in the nivo3 and ipi1 group was 42 weeks and the median OS had not yet been reached. This dose combination appeared to be no less active but less toxic than nivolumab 1 mg/kg plus ipilimumab 3 mg/kg, while nivolumab 3 mg/kg plus ipilimumab 3 mg/kg (nivo3 and ipi3) proved unacceptably toxic in this population. The nivo3 and ipi3 arm (*n*=6) therefore did not proceed to expansion [98]. Updated results with 2-year follow-up reported ORR of 40% in both treatment arms (nivo3 and ipi1, nivo1 and ipi3) and median DOR of 20.4 weeks (nivo3 and ipi1, *n*=47) and 19.4 weeks (nivo1 and ipi3, *n*=47) [99].

Checkmate 214 compares the nivo3 and ipi1 regimen with sunitinib in a phase III trial in advanced ccRCC. The trial plans to recruit 1070 patients with locally advanced or mRCC, previously untreated with any systemic therapy and randomize them between sunitinib and nivolumab and ipilimumab (nivo3 and ipi1) every 3 weeks for four treatments following by nivolumab 3 mg/kg every 2 weeks until progression. Primary results are expected in late 2017 [99].

### Combination regimens – immune checkpoint inhibitors and angiogenesis inhibitors

Encouraging early results from JAVELIN Renal 100, a phase 1b, dose-finding study, demonstrated durable responses in six of six treatment-naïve patients evaluable for response to avelumab (MSB0010718C), a PD-L1 inhibitory mAb, given in combination with axitinib. The trial aims to recruit up to 55 patients, who will receive avelumab (10 or 5 mg/kg, every 2 weeks) plus axitinib (5 or 3 mg twice daily) in dose-finding and dose-expansion cohorts [114]. A follow-on phase III trial of avelumab plus axitinib compared with sunitinib monotherapy as first-line treatment of aRCC is also underway [115].

In addition to the trials listed in Table 2, the combination of pembrolizumab plus pazopanib was investigated in a phase I/II trial and unfortunately results presented at the American Society of Clinical Oncology (ASCO) Conference in June 2017 deemed it unsafe and reported significant concerns regarding hepatotoxicity. After a dose-escalation phase, 20 patients were enrolled in expansion cohorts. The combination regime resulted in 90% of patients reporting G3/4 AEs; resulting in 50% of patients permanently discontinuing treatment and 80% requiring dose interruptions or reductions. The combination did exhibit early antitumour efficacy however the toxicity profile was intolerable. When sequential therapy was explored, other G3/4 AEs developed including diarrhoea, increased amylase, perforation of the large intestine, pneumonitis and confusion [116]. CALYPSO is a phase I/II clinical trial investigating durvalumab (an anti-PD-L1 mAb) in combination with tremelimumab (anti-CTLA-4 mAb) and/or savolitinib (AZD6094; a highly selective MET TKI) in ccRCC and papillary RCC. The trial aims to recruit 195 patients with the estimated study completion date in September 2019 [117]. Pembrolizumab plus axitinib is currently being investigated in KEYNOTE-426 a phase III trial in the first-line setting, with the standard of care comparator arm as sunitinib [118]. The combination of lenvatinib (a multitarget TKI) with pembrolizumab is being compared in a three-arm phase III trial with lenvatinib plus everolimus or sunitinib in the first-line setting [119].

Atezolizumab is currently under evaluation in IMmotion 151, a phase III clinical trial with bevacizumab. The trial plans to recruit 900 patients with aRCC (sarcomatoid or ccRCC.) Patients are randomized to atezolizumab plus bevacizumab, atezolimuzab or sunitinib in the first-line setting. Preliminary results have reported responses in 25–46%. Median PFS was highest in the subgroup who were PD-L1-positive on tumour testing [120].

## Adjuvant trials

ADAPTeR is a phase II adjuvant study currently ongoing, where nivolumab is administered as pre- and post-operative therapy in mRCC [121]. Pembrolizumab is similarly being investigated with patients (planned accrual, *n*=36) proposed to receive pembrolizumab every 3-weeks for up to three cycles followed by standard of care surgical resection; and then may receive post-resection pembrolizumab every 3 weeks for up to 1 year (17 cycles). In the alternate experimental arm, patients will undergo surgical resection then commence pembrolizumab for up to 1 year (17 cycles) [122]. At present, there are no adjuvant immunotherapy phase III trials in recruitment.

## nccRCC

This review does not cover in depth nccRCC. A phase II clinical trial, Keynote 427, is currently recruiting both ccRCC and a prespecified cohort of nccRCC patients to treatment with pembrolizumab every 3 weeks for up to 35 doses (approximately 24 months) [123]. SUNIFORECAST is a randomized phase II trial comparing ipilimumab plus nivolumab with sunitinib in the non-clear cell population, which is ongoing [124].

## Conclusion

Nivolumab has been rapidly adopted into the routine care of patients with aRCC who have failed prior therapy where it has proven a magnitude of clinical benefits not seen with previous systemic therapies in this disease. Emerging data, including the prolonged DOR observed with this intervention and experiences with similar drugs in other diseases, suggest that, for some patients with incurable mRCC, immune checkpoint inhibition may present the opportunity of long term survival. Current trials should help maximize these benefits by bringing therapy into the front-line setting as an alternative or a complement to TKIs, and to explore their benefits in the adjuvant setting where there is the prospect of increasing the cure rate from surgery. Future research focuses on the discovery and development of newer and better ways of manipulating the immune system for therapeutic gain and on finding ways of better stratifying patients to select and prioritize these treatments for those where they will offer the maximum gain.

## Abbreviations

AE: adverse event
ALT: alanine transaminase
AST: aspartate transaminase
APC: antigen-presenting cell
CAIX: carbonic anhydrase IX
ccRCC: clear cell renal cell cancer
CR: complete response
CRC: colorectal cancer
CTL: cytotoxic T lymphocyte
CTLA-4: cytotoxic T lymphocyte associated protein 4
DC: dendritic cell
DOR: duration of response
EMA: European Medicines Agency
FDA: Food and Drug Administration
HD: high dose
HIF: hypoxia-inducible factor
IFN-α: interferon-α
IFN-γ: interferon-γ
IL-2: interleukin-2
irAE: immune-related AE
LDH: lactate dehydrogenase
mAb: monoclonal antibody
mRCC: metastatic renal cell cancer
MSKCC: Memorial Sloan Kettering Cancer Center
mTOR: mammalian target of rapamycin
nccRCC: non-clear cell renal cell cancer
NK: natural killer
NSCLC: non-small-cell lung cancer
ORR: overall response rate
OS: overall survival
PD-1: programmed death-1
PD-L1: programmed death-ligand 1
PFS: progression-free survival
PTEN: phosphatase and tensin homologue
QoL: quality of life
RCC: renal cell cancer
SNP: single nucleotide polymorphism
TAM: tumour-associated macrophage
TCR: T-cell receptor
TIL: tumour-infiltrating lymphocyte
TKI: tyrosine kinase inhibitor
TNF: tumour necrosis factor
Treg cell: regulatory T cell
VEGF: vascular endothelial growth factor
VHL: von Hippel–Lindau
